# Identification of Rubisco *rbcL* and *rbcS* in *Camellia oleifera* and their potential as molecular markers for selection of high tea oil cultivars

**DOI:** 10.3389/fpls.2015.00189

**Published:** 2015-03-31

**Authors:** Yongzhong Chen, Baoming Wang, Jianjun Chen, Xiangnan Wang, Rui Wang, Shaofeng Peng, Longsheng Chen, Li Ma, Jian Luo

**Affiliations:** ^1^National Engineering Technology Research Center of Oil-tea Camellia, Hunan Academy of ForestryChangsha, China; ^2^Environmental Horticulture Department and Mid-Florida Research and Education Center, University of FloridaApopka, FL, USA

**Keywords:** *Camellia oleifera*, camellia oil, photosynthesis, Rubisco, *rbcL*, *rbcS*, tea oil

## Abstract

Tea oil derived from seeds of *Camellia oleifera* Abel. is high-quality edible oil in China. This study isolated full-length cDNAs of Rubisco subunits *rbcL* and *rbcS* from *C. oleifera*. The *rbcL* has 1,522 bp with a 1,425 bp coding region, encoding 475 amino acids; and the *rbcS* has 615 bp containing a 528 bp coding region, encoding 176 amino acids. The expression level of the two genes, designated as *Co-rbcL* and *Co-rbcS*, was determined in three *C. oleifera* cultivars: Hengchong 89, Xianglin 1, and Xianglin 14 whose annual oil yields were 546.9, 591.4, and 657.7 kg ha^-1^, respectively. The *Co*-*rbcL* expression in ‘Xianglin 14’ was significantly higher than ‘Xianglin 1’, and ‘Xianglin 1’ was greater than ‘Hengchong 89’. The expression levels of *Co-rbcS* in ‘Xianglin 1’ and ‘Xianglin 14’ were similar but were significantly greater than in ‘Hengchong 89’. The net photosynthetic rate of ‘Xianglin 14’ was significantly higher than ‘Xianglin 1’, and ‘Xianglin 1’ was higher than ‘Hengchong 89’. Pearson’s correlation analysis showed that seed yields and oil yields were highly correlated with the expression level of *Co-rbcL* at *P* < 0.001 level; and the expression of *Co-rbcS* was correlated with oil yield at *P* < 0.01 level. Net photosynthetic rate was also correlated with oil yields and seed yields at *P* < 0.001 and *P* < 0.01 levels, respectively. Our results suggest that *Co-rbcS* and *Co-rbcL* in particular could potentially be molecular markers for early selection of high oil yield cultivars. In combination with the measurement of net photosynthetic rates, the early identification of potential high oil production cultivars would significantly shorten plant breeding time and increase breeding efficiency.

## Introduction

**FIGURE 1:**
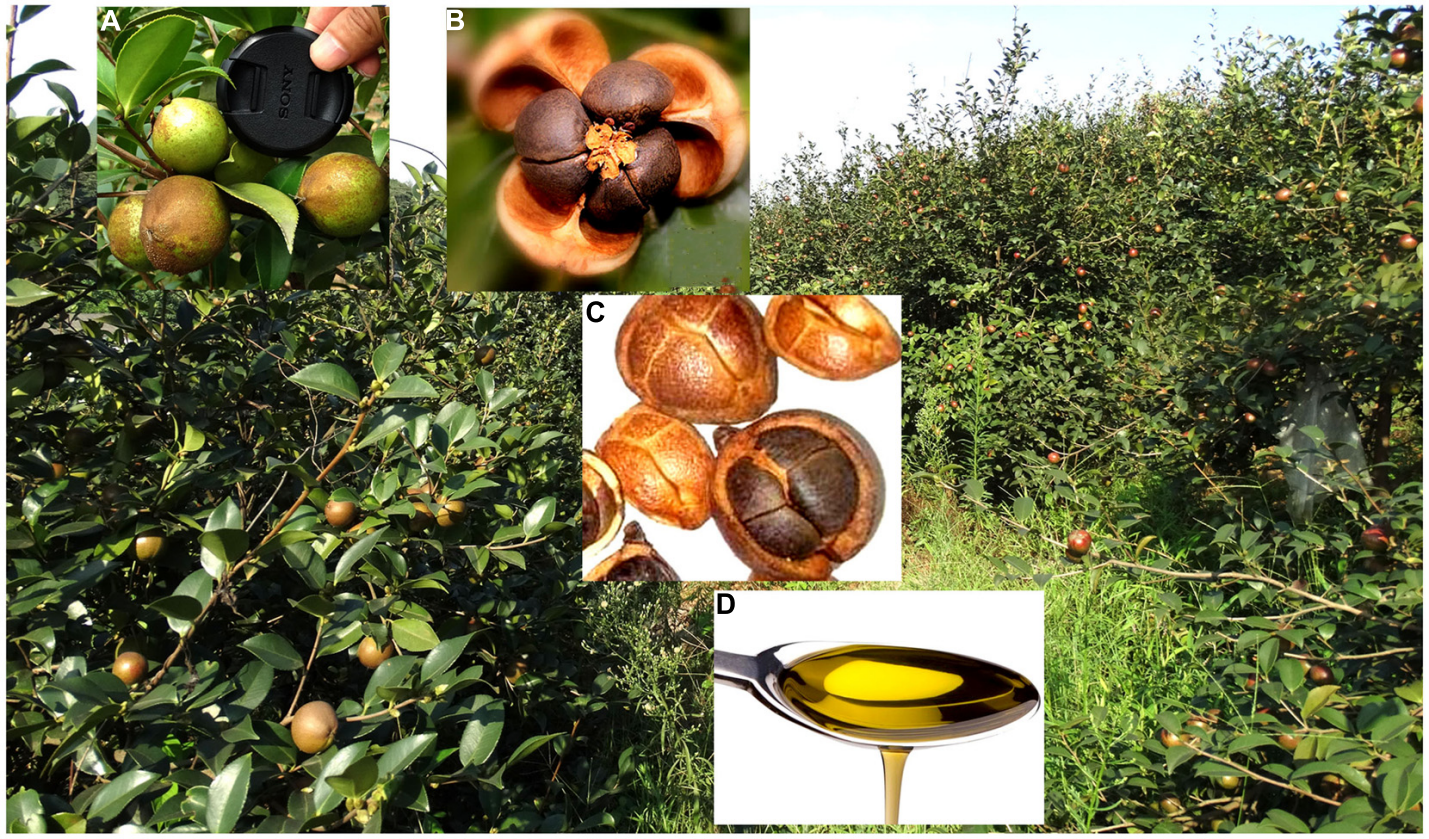
*Camellia oleifera* “Xianglin 14’ produced abundant fruit. Fruit diameters were up to 4 cm **(A)**; seed appearance at seed shell opening **(B)**; a mix of seeds, nutlets, and fruit **(C)**; and tea oil derived from seeds **(D)**.

*Camellia oleifera* Abel. is a shrub or small tree native to China (Mondal, 2011). It has been cultivated in south-central and southern China for more than 2,000 years primarily for edible oil extracted from seeds (Wei et al., 2012; **Figure 1**). Commonly known as tea oil or camellia oil, it is comprised of 67.7–76.7% oleic acid, 82–84% unsaturated fatty acids, 68–77% monounsaturated fatty acids, and 7–14% polyunsaturated acid, which is similar to the composition of olive oil (Ma et al., 2011). Additionally, tea oil has been used for a wide range of cosmetic and medicinal purposes (Lee and Yen, 2006; Cheng et al., 2014). There has been increasing demand for producing camellia oil in China (Wei et al., 2012; Liao et al., 2014).

The production of *C. oleifera* increased to more than 3.7 million hectares in China (Kong et al., 2013), however, the increased production area still did not meet the demand for tea oil. This is largely due to conventional cultivars having low oil yield ranging from 105 to 225 kg ha^-1^ (Zhuang, 2008). To improve tea oil yield, the National Engineering and Technology Research Center of *Camellia* Oil at the Hunan Academy of Forestry in Changsha, Hunan, China initiated a breeding program focusing on germplasm evaluation and new cultivar development of *C. oleifera*. To date, more than 20 new cultivars have been developed. Tea oil yield of the new cultivars ranged from 450 to 750 kg ha^-1^ (Zhuang, 2008).

*Camellia oleifera* is self-sterile, which is caused by prezygotic late-acting self-incompatibility (Liao et al., 2014). Thus, all cultivars are genetically highly heterozygous. Methods currently used for *C. oleifera* breeding include conventional hybridization and selection of individuals from progenies with high oil content. Because *C. oleifera* will neither flower and nor bear fruit in the first 4–5 years, full bloom and seed production occurs in 5–7 years. As a result, breeding of *C. oleifera* is a long and time-consuming process, requiring 7–10 years to develop a new cultivar. After multiple regional trials, new cultivars are propagated vegetatively through grafting or rooting of cuttings. The propagated plants require additional 2–4 years to reach the fruiting stage; therefore, there is a critical need for improving breeding efficiency of *C. oleifera*.

Marker-assisted selection is an indirect selection method in which a trait of interest is selected based not on the trait itself, but on a marker related to it (Collard and Mackill, 2008). The assumption is that the marker used for trait selection associates at high frequency with the genes of interest. Due to the genetic linkage, marker-assisted selection is particularly useful for traits that are expressed late in development. Recently, a *C. oleifera* EST library was constructed (Tan et al., 2006), and microsatellite markers for the genus *Camellia* were also reported (Qiang et al., 2013). However, there are still no morphological, cytological, or molecular markers related to oil yield in *C. oleifera*, and no any markers have been used for its breeding.

Ribulose 1, 5-bisphosphate carboxylase/oxygenase (EC 4.1. 1.39, Rubisco) should be an important biochemical marker as it can comprise up to 50% of the total soluble protein in plant leaves (Ellis, 1979). Rubisco is the key regulatory enzyme catalyzing CO_2_ fixation and ribulose diphosphate oxygenase reaction dual function, which determines net photosynthesis (Andersson and Backlund, 2008). The holoenzyme of Rubisco is composed of eight small subunits (SSU, 14–15-kDa) encoded by a nuclear multigene family (*rbcS*), and eight large subunits (LSUs, 52-kDa) encoded by a single gene (*rbcL*) in the multicopy chloroplast genome. SSU precursors are processed during entry into the chloroplast and then assembled with the LSUs to yield the Rubisco holoenzyme (Ichikawa et al., 2008). The *rbcL* gene is highly conserved, while *rbcS* is less conserved (Manzara and Gruissem, 1988). Recent molecular phylogenetic analysis, however, revealed that positive selection in the *rbcL* gene of terrestrial land plants is quite a common phenomenon (Kapralov and Filatov, 2007; Galmes et al., 2014). Since plant breeding is considered a man-made evolution (Jauhar, 2006; Ghai, 2009), we assumed that long-term selection of *C. oleifera* cultivars for high oil yield could lead to enrichment in the abundance of transcript of Rubisco genes for enhancing photosynthetic efficiency and increasing oil biosynthesis. This is based on the notion that the reducing power, ATP, and a range of metabolites derived from photosynthesis support high rates of fatty acid synthesis (Rawsthorne, 2002; Bates et al., 2013). Thus, Rubisco subunit genes could be molecular markers for improving breeding efficiency. Rubisco *rbcL* and *rbcS* genes, however, have not been isolated from *C. oleifera*. Furthermore, since Rubisco activity is directly related to photosynthesis, it is possible that net photosynthetic rate could be a physiological marker for detecting seed yield and increased oil production.

The objectives of this study were to (1) clone and identify *rbcL* and *rbcS* genes from *C. oleifera*; (2) analyze *rbcL* and *rbcS* expression in three cultivars that differed in seed and oil yields; (3) examine net photosynthetic rates of the three cultivars; and (4) determine if there were any relationships among *rbcL* and *rbcS* gene expressions, net photosynthetic rates, and oil yield.

## Materials and Methods

### Plant Materials and Growth Conditions

Three cultivars Hengchong 89, Xianglin 1, and Xianglin 14 were planted in 2006 in field plots of the Experimental Station of the National Engineering and Technology Research Center of Oil-Tea *Camellia*, Hunan Academy of Forestry, Changsha, Hunan, China, which is located between 111° 53′–114° 05′ E longitude and 27° 51′–28° 40′ N latitude. Annual average temperature is 17.03°C, with a mean of 4.6°C in January and 29.0°C in July. Average annual precipitation is 1,331 mm, and a maximum photosynthetic photon flux density of 2,000 mmol m^-2^ s^-1^. Hengchong 89 is a popular cultivar in Hunan and other south-central provinces, and Xianglin 1 and Xianglin 14 were developed by the Center of Oil-Tea *Camellia*. Hengchong 89, a well-established cultivar, was a control. Xianglin 1 and Xianglin 14, due to their adaptation to the climate of south-central China and drought resistance, have been increasingly grown in this region. Three plants of each cultivar were randomly planted in experimental plots that were 54 m^2^ each, and plants were spaced at 2.0 m × 3.0 m. There were three plots, and each plot represented a block. Plants produced fruit in 2010. Starting from 2011, fruits were harvested and weighed annually.

### Extraction of DNA and RNA and Synthesis of first Strand cDNA

The second leaf from the apical meristem was harvested, frozen in liquid nitrogen, and stored in a freezer (-80°C). The total DNA was extracted from the leaves using plant genomic DNA extraction kit (Tiangen Biotech Co., Beijing, China) for isolation of *rbcL*.

Two sets of total RNA were isolated: one from three cultivars Hengchong 89, Xianglin 1, and Xianglin 14 and the other from Xianglin 14. Frozen tissues were ground in liquid nitrogen and lysed with 600 μl 3x CTAB plus 1% 2-mercaptoethanol, followed by E.Z.N.A.TM Plant RNA Kit Reagent (OMEGA, USA). The RNase-free DNase (OMEGA, USA) was used to remove any remaining contaminating DNA from the total RNA extractions. An aliquot of each sample was checked on an agarose gel. The concentration and purity of each RNA sample was determined using Nanodrop2000 (Thermo scientific, USA).

For isolation of *rbcS*, total RNA (500 ng) from the leaf of Xianglin 14 was used to generate a single-stranded cDNAs using an oligo (dT)_18_ primer and M-MLV reverse transcriptase in the presence of an RNase inhibitor (Thermo Scientific, USA) in a volume of 20 μl.

### Cloning and Identification of *rbcL* and *rbcS* from *C. oleifera*

To clone *rbcL*, forward and reverse primers 5′-GGGAGGGACTTATGTCACCA-3′ and 5′-TGTATTCGGCTCAATCCTTT-3′ were designed in reference to the *rbcL* nucleotide sequences from *Arabidopsis thaliana* (GenBank access number: ATU91966), *Brassica napus* (AF267640), and *Glycine max* (EU717256 and Z95552) using Primer Premier 5.0. Polymerase chain reaction (PCR) was carried out in a 20 μl reaction containing 12.5 μl PrimeSTAR buffer, 0.5 mM dNTPs, 1.5 mM MgCl_2_, 0.5 mM of each primer, 1 U PrimeSTAR HS DNA Polymerase (Takara, Dalian, China), and 20 ng of the total genomic DNA as a template. Cycling conditions consisted of pre-cycling at 98°C for 2 min, and 36 cycles of denaturation at 98°C for 40 s, annealing at 54.5 °C for 40 s, elongation at 72°C for 2 min, and an elongation phase at 72°C for 7 min. The PCR products were analyzed on 1.2% (w/v) agarose gel, purified using a gel extraction mini kit, and ligated into pMD18-T vector (Takara, Dalian, China). The mixtures were transformed into *Escherichia coli* strain DH5α, and the positive clones were sequenced (data not shown).

For cloning of *rbcS*, a partial cDNA fragment was cloned by degenerate PCR using reverse transcription polymerase chain reaction (RT-PCR). Degenerate primers of *rbcS* 5′-AAGAAGTTYGAGACSCTSTC-3′ (forward) and 5′-GGCWTGWAGGCGATGAAYCTG-3′ (reverse) were designed using Primer Premier 5.0. PCR was performed in a 20 μl reaction containing 1× PCR buffer, 0.5 mM dNTPs, 1.5 mM MgCl_2_, 0.5 mM of each degenerate primer of *rbcS*, 1 U Taq polymerase, and 2.0 μl of the cDNA first strand. Cycling conditions consisted of pre-cycling at 94°C for 5 min followed by 35 cycles at 94°C for 30 s, 53°C for 30 s, and 72°C for 2 min. The reaction was terminated by a 7-min incubation step at 72°C. Rapid amplification of cDNA ends (RACE) was performed by using reagents of 5′ and 3′ RACE purchased from Invitrogen (Carlsbad, CA, USA), and conducted according to their instruction manual.

Gene specific oligonucleotide primers (GSPs) for *rbcS* 5′ RACE and 3′ RACE were designed as: *rbcS* 5′ RACE GSP1: 5′-CCTCAACCTCCTTCAACA-3′; *rbcS* 5′ RACE GSP2: 5′-TCCAGTATCGCCCATCGTAG-3′; and *rbcS* 5′ RACE GSP3: 5′-CACAAATCCTCCCACAACAG-3′. GSPs for *rbcS* 3′ RACE were as follows: *rbcS* 3′ RACE GSP1: 5′-ATGGGCGATACTGGACAATG-3′; *rbcS* 3’RACE GSP2: 5′-GTGTTGAAGGAGGTTGAGGA-3′; and *rbcS* 3′RACE GSP3: 5′-AGAAGGAATACCCACAAGCA-3′. *rbcS* 5’RACE was obtained in a 25 μl reaction containing 12.5 μl 2 × MightyAmp Buffer including Mg^2+^ 4 mM (2x), dNTP 800 μM (2x); UPM (long); *rbcS* 5′ RACE GSP 0.75 μl (0.3 μM); the synthesized cDNA as template; and Mighty Amp DNA polymerase 0.5 U (1.25 U/μl). Cycling conditions consisted of pre-cycling at 98°C for 2 min, and 35 cycles of denaturation at 98°C for 40 s, annealing at 54°C for 40 s, and elongation at 68°C for 2 min, and final extension at 72°C for 7 min. For the full-length *rbcS* gene, the PCRs were performed with the forward and reverse primers 5′-ATGGTTGCCTCCATAC-3′ and 5′-CACAGCCATTGATCTAACGA-3′ in a 20 μl reaction containing PrimeSTAR buffer 12.5 μl, 0.5 mM dNTPs, 1.5 mM MgCl_2_, 0.5 mM of each primer, 1 U PrimeSTAR HS DNA Polymerase, and 2.0 μl cDNA first strand as template. Cycling conditions consisted of pre-cycling at 98°C for 2 min, and then 35 cycles of denaturation at 98°C for 40 s, annealing at 51°C for 40 s, elongation at 72°C for 2 min, and an elongation phase at 72°C for 7 min. The PCR products were separated, purified, ligated, transformed, and sequenced as described above.

### Analysis of Sequence Properties

Database was retrieved using the NCBI server^1^, and the protein properties were analyzed using ProtParam^2^, TMpred^3^, and SignalP 3.0 Server^4^. The secondary structures were predicted by SOPMA^5^. The homolog relationships between deduced amino acid sequences of *rbcL* and *rbcS* from *C. oleifera* and other higher plants were analyzed by Vector NTI and displayed by Gendoc. The subcellular location and cleavage site analysis were conducted using the ChloroP 1.1 prediction server^6^.

### Transcript Abundance Analysis by Quantitative RT-PCR

Equal volumes of RNA solution isolated from leaves of three cultivars were used to synthesize single-stranded cDNA in a 20 μl volume using Transcriptor First Strand cDNA Synthesis Kit (Thermo Scientific, USA) with anchored oligo(dT)_18_ primers. Aliquots of the single-stranded cDNA of the three cultivars were used as templates for qRT-PCR analysis on CFX96 (Bio-Rad, Hercules, CA, USA). PCR amplifications were performed in a total volume of 20 μl using the Maxima SYBR Green qPCR Master Mix (2x) (Thermo Scientific, USA) and the specific primers designed for *rbcL* of *C. oleifera* were 5′-TGTACTACAGTTCGGCGGAG-3′ (forward), and 5′-TCCATACCTCACAAGCAGCA-3′ (reverse); for *rbcS* of *C. oleifera,* were 5′-TGGGCGATACTGGACAATGT-3′ (forward), and 5′-CAGGCGATGAAACTGATGCA-3′ (reverse). The internal control was *GAPDH* (glyceraldehyde-3-phosphate-dehydrogenase) gene. The primers of *GAPDHF*: 5′-GAAGGGTGGTGCAAAGAAGG-3′ and *GAPDHR* 5′-GACCCTCAACAATGCCAAACT-3′ were designed using Primer 3^7^. The sizes of these amplicons were 194, 165, and 185 bp, respectively.

### Photosynthesis Measurement

Net photosynthetic rates (*P_N_*) of the three cultivars were determined using a Li-6400 portable photosynthesis meter (Li-COR Bioscience, Lincoln, NE, USA). Other parameters including stomatal conductance (*g*_s_), intercellular CO_2_ concentration (*C*i), and transpiration rate (*E*) were also recorded. The measurements took place on the newest mature leaves at 11:00 am–12:00 pm, four leaves per plant and three readings per leaf. Since each block had nine plants, three plants per cultivar and there were three blocks; a total of nine plants were measured per cultivar in every September from 2011 to 2013. The environmental conditions for the measurements were set as follows: leaf temperature at 25°C, photon flux density at 1,000 μmol m^-2^ s^-1^, relative humidity at 65%, and CO_2_ concentration at 0.04%.

### Data Analysis

The experiment for oil production of the three cultivars was arranged as a randomized complete block design with three replications. Fresh fruit was harvested annually from 2011 to 2013; fresh seeds and dry seeds per plant were recorded. Fresh and dry seed yields were calculated based on 1,650 plants per hectare. Tea oil was extracted using the Soxhlet extraction method described by Zeng et al. (2014). Oil yield was calculated based on the amount of oil extracted from seeds.

Data of seed yields, oil yields, and photosynthetic parameters collected in 2011, 2012, and 2013 were analyzed annually (data not shown). The 3-year data were combined based on parameters and analyzed by analysis of variance (SAS GLM, SAS Institute, Cary, NC, USA); mean separations were performed using Duncan’s Multiple Range Test at the 5% level.

The quantitative RT-PCR data were analyzed using the Bio-Rad CFX 2.0 data analysis software. The expression levels of *Co*-*rbcL* and *Co-rbcS* from *C. oleifera* were normalized based on the internal control of *GAPDH* gene.

Pearson’s correlation analysis was performed to assess the relationship among seed and oil yields, *Co*-*rbcL* and *Co-rbcS* expressions, and *P_N_*. The significance of the correlations was tested using the critical value table (Taylor and Bates, 2013).

## Results

### Seed and Oil Yields of Three Cultivars

**FIGURE 2:**
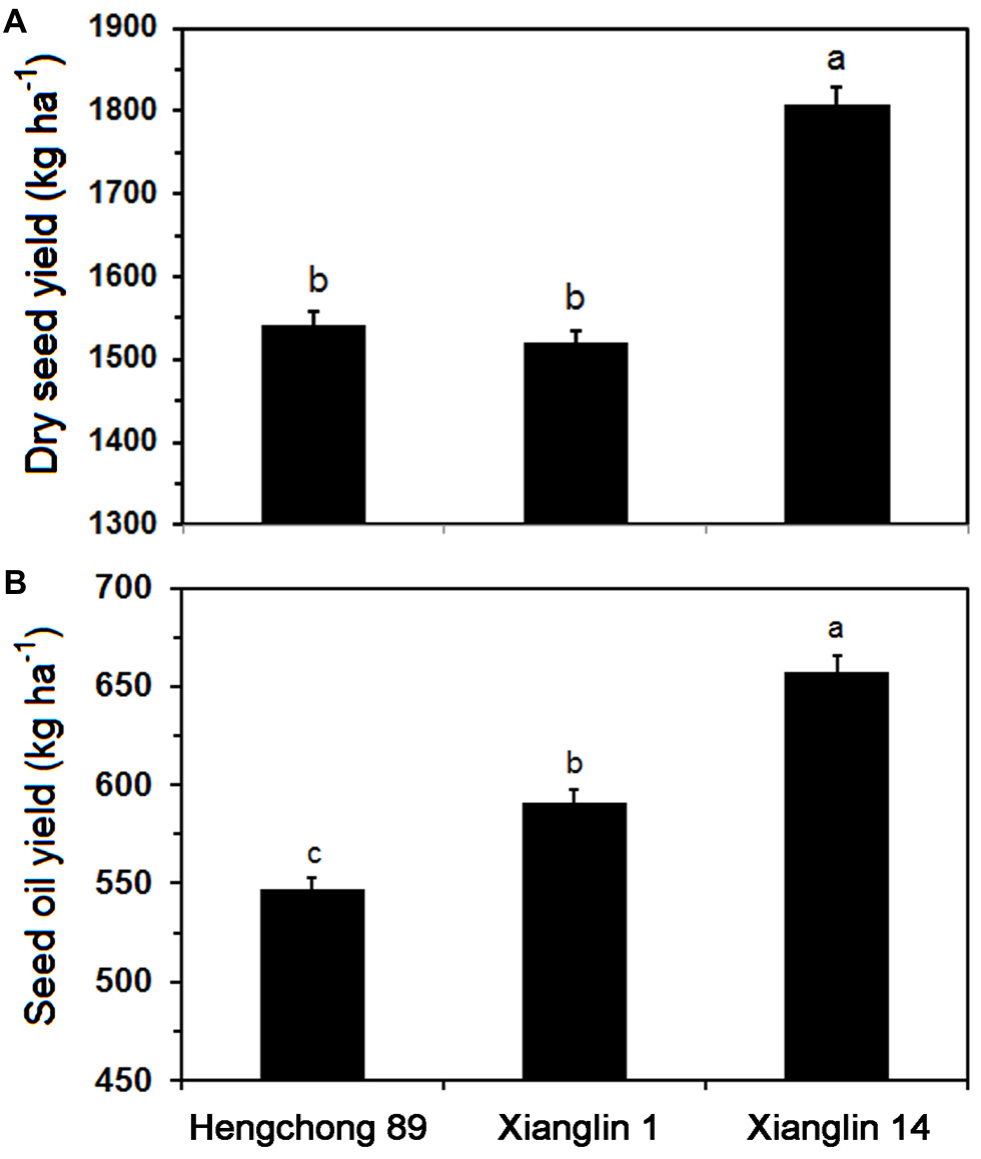
Mean dry seed **(A)** and oil **(B)** yields of three cultivars Hengchong 89, Xianglin 1, and Xianglin 14 grown at the Experimental Station of the National Engineering and Technology Research Center of Oil-Tea *Camellia*, Changsha, Hunan, China from 2011 to 2013. Bars represent standard error with *n* = 9. Different letters above the bar represent significant difference at *P* < 0.05 level by Duncan’s Multiple Range Test.

Dry seed yields of three cultivars from 2011 to 2013 averaged 1,540.7, 1,520.3, and 1,806.9 kg ha^-1^ annually for Hengchong 89, Xianglin 1, and Xianglin 14, respectively (**Figure 2A**). The seed yield of Xianglin 14 was significantly higher than that of Hengchong 89 and Xianglin 1. Oil extracted from the seeds or oil yield of Xianglin 14 was 657.7 kg ha^-1^, which was significantly greater than 591.4 kg ha^-1^ produced by Xianglin 1 (**Figure 2B**), and oil yield of Xianglin 1 was higher than Hengchong 89 at 546.9 kg ha^-1^.

### Cloning and Identification of *rbcL* and *rbcS* cDNAs from *C. oleifera*

**FIGURE 3:**
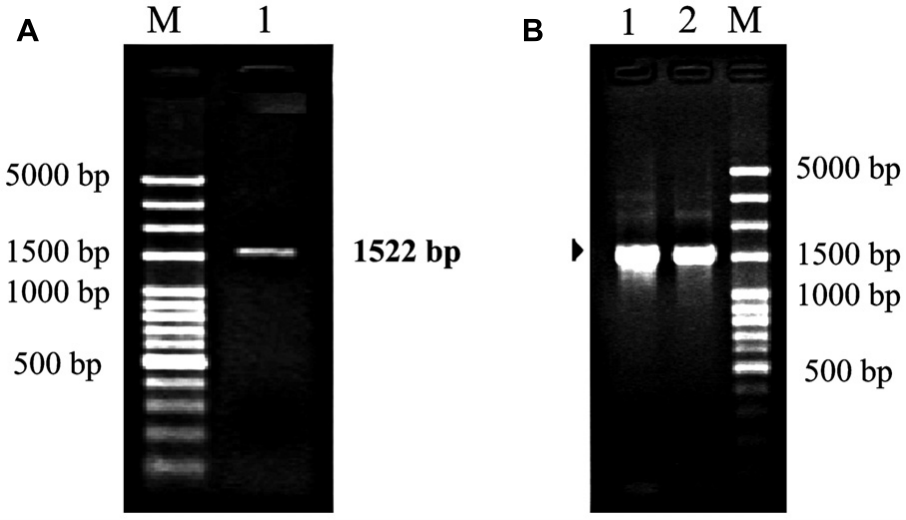
Polymerase chain reaction cloning of *rbcL* from leaves of *C. oleifera* and its recombinant strains. **(A)** Lane 1: the PCR amplification fragment of *rbcL* with an expected band at 1,522 bp for *rbcL* amplified by PCR. **(B)** Lanes 1 and 2 represent the positive recombinant strains. M represents 100 bp plus DNA ladder.

Polymerase chain reaction amplification generated a product about 1,500 bp (**Figure 3**). After sequencing, the product was confirmed to be 1,522 bp containing a 1,425 bp coding region and encoding 475 amino acids with a molecular mass of 52.63 kDa. The nucleotide sequence was 99% homologous with that from other *Camellia* species, such as *Camellia sinensis* (GenBank accession number: KC143082), *Camellia japonica* (L12602), and *Camellia granthamiana* (AF380034). Such a high homology suggested that this fragment from *C. oleifera* was *rbcL* gene, which was designated as *Co-rbcL*. The sequence has been deposited in GenBank with an accession no. KJ721197.

**FIGURE 4:**
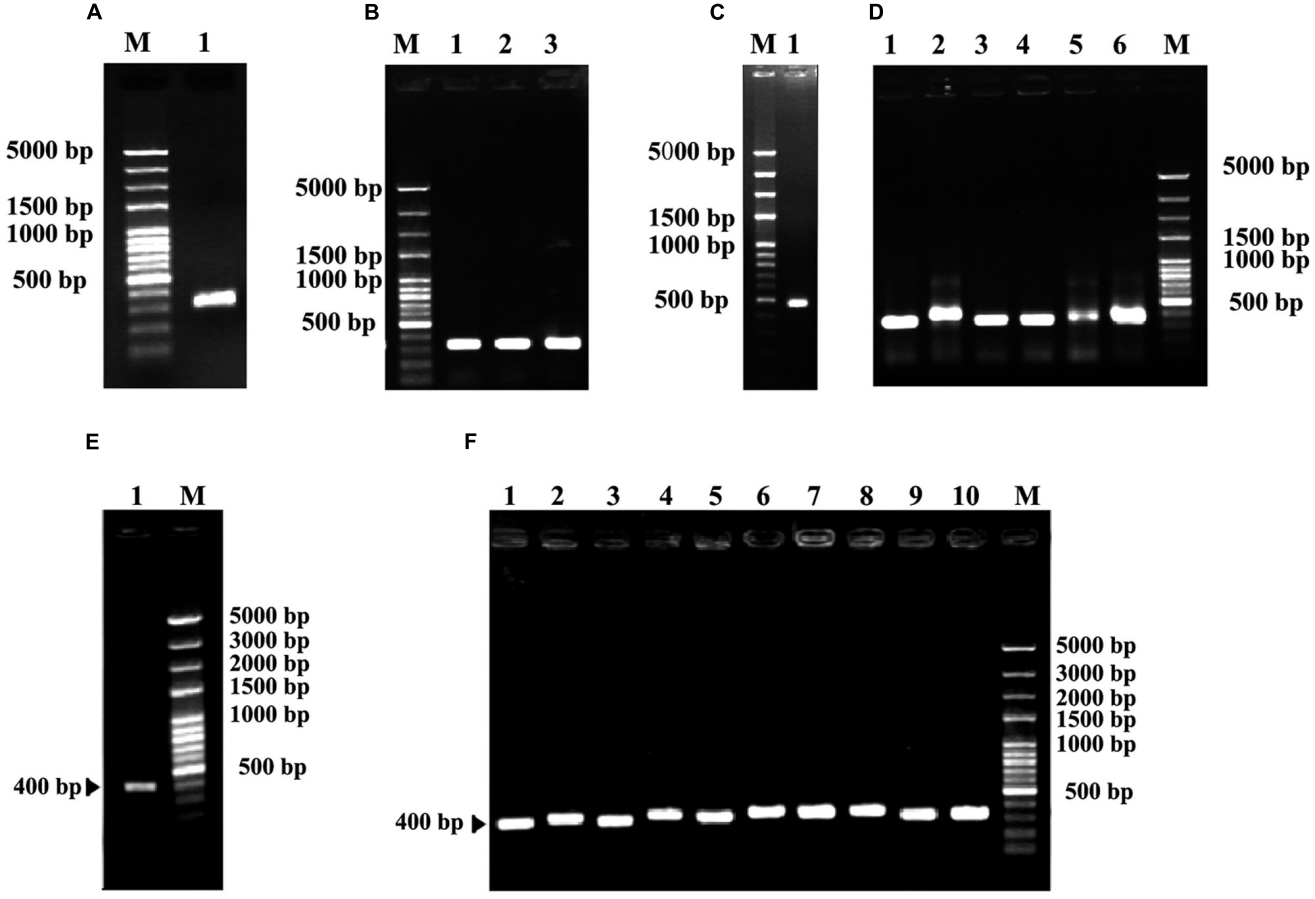
Polymerase chain reaction cloning of *rbcS* from leaves of *C. oleifera* and its recombinant strains. **(A)** The degenerate PCR amplification fragment of *rbcS* in lane 1. **(B)** Lanes 1, 2, and 3 are positive recombinants of *rbcS*. **(C)** Lane 1 indicates the 5′ RACER result of *Co-rbcS*. **(D)** Lanes 1–6 were positive recombinants of the 5′ RACER of *Co-rbcS*. **(E)** Lane 1 is the 3′ RACER result of *Co-rbcS*. **(F)** Lanes 1–10 are positive clones of *Co-rbcS*. M represents 100 bp plus DNA ladder.

A partial sequence of *rbcS* gene was first obtained from degenerate RT-PCR reactions. Sequence analysis showed that this fragment had 324 bp (**Figures 4A,B**) encoding a deduced peptide sequence of 122 amino acids. This sequence shared 94% homology with *rbcS* of *C. sinensis* (GenBank accession no. EF011075), suggesting that it was a fragment of *rbcS* gene. Based on this fragment, a set of gene specific primers were designed in order to obtain the 5′ and 3′ ends (**Figures 4C–F**). The resultant full-length *rbcS* gene was found to be 615 bp. This sequence had an open reading frame of 528 bp starting with an initiation codon ATG at position of the 2^nd^ and 4^th^ nucleotides and ending with a termination codon TAA at position of the 530^th^ and 532^nd^ nucleotides, encoding 176 amino acid residues with a molecular mass of 19.69 kDa. The GC content of this sequence was about 49.15%. The predicted nucleotide sequence of the coding region shared more than 95% homology with that of *rbcS* mRNA from *C. sinensis* (GenBank accession no. EF011075). This sequence was designated as *Co-rbcS*, and has been deposited in GenBank with an accession no. KJ721196.

### Characteristics of Co-rbcL and Co-rbcS Proteins

**FIGURE 5:**
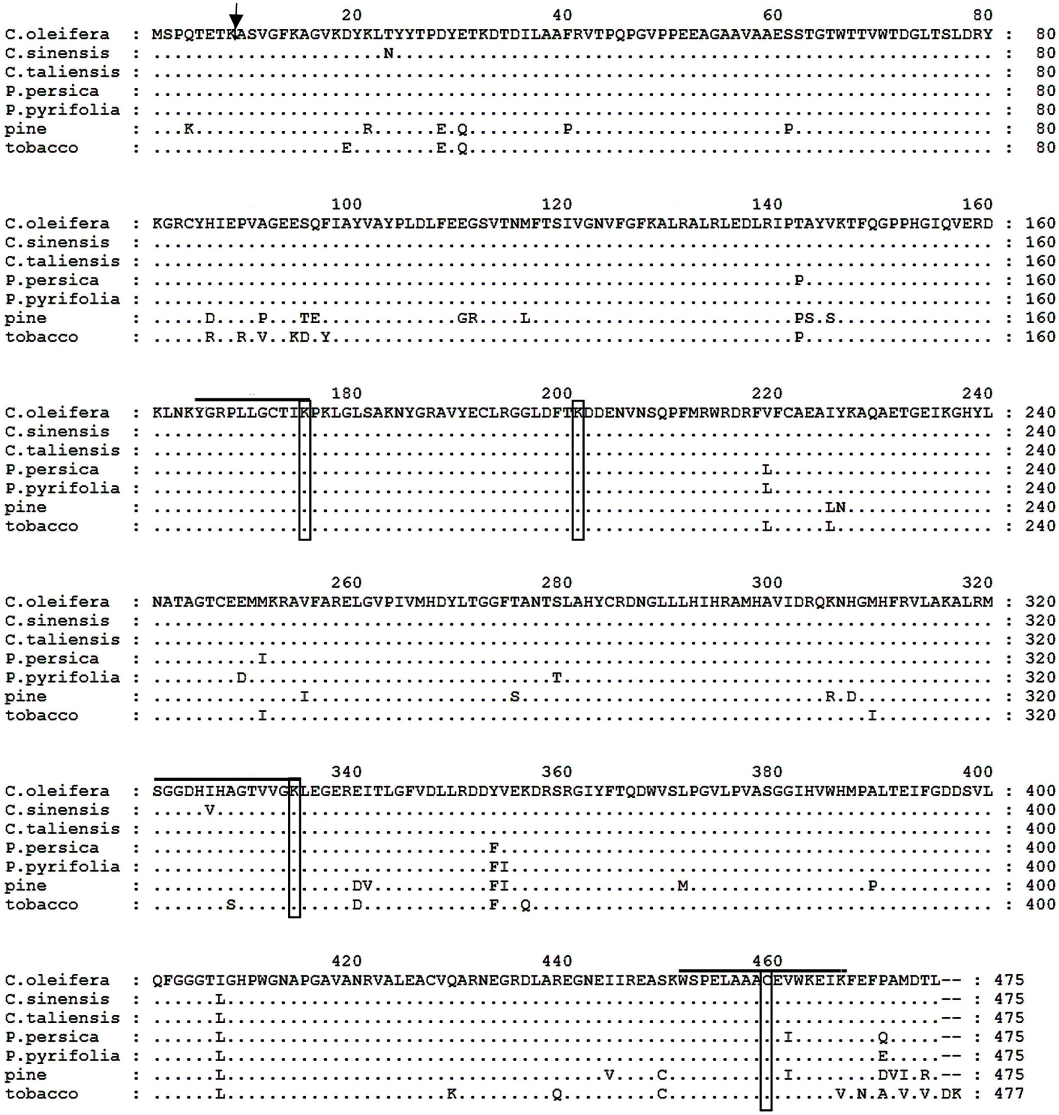
Comparison of the deduced amino acid sequences of the rbcL protein from *Camellia oleifera*, *Camellia sinensis*, *Camellia taliensis*, *Prunus persica*, *Pyrus pyrifolia*, pine (*Pinus thunbergii*), and tobacco (*Nicotiana tabacum*). Amino acid residues identical to those of *C. oleifera* rbcL protein are indicated by dots. Hyphens indicate gaps. The arrow indicates the possible processing site of mature protein in *C. oleifera*; and active site residues are overlined and boxed.

The putative Co-rbcL amino acid sequence shared an overall homology of 100, 99, 98, 98, 94, and 92% with that of *Camellia taliensis* (Yang et al., 2013), *C. sinensis* (GenBank accession no. YP_007317256), peach (*Prunus persica*, GenBank accession no. YP_004021673), *Pyrus pyrifolia* (GenBank accession no. YP_004842247), tobacco (*Nicotiana tabacum*; Shinozaki and Sugiura, 1982), and pine (*Pinus thunbergii*; Mukai et al., 1991), respectively (**Figure 5**). Noticeably, there were active sites residing in these aligned sequences, including ^165^YGRPLLGCTIKPK^177^, K-177, ^321^SGGDHI/VHAGTVVGKLEGER^339^, K-334, K-201, and C-459 (Mukai et al., 1991). The Co-rbcL processing site was postulated to occur between the Lys(K)^51^ and Arg(A)^9^ of the protein sequence using the ChloroP 1.1 Prediction Server^6^.

**FIGURE 6:**
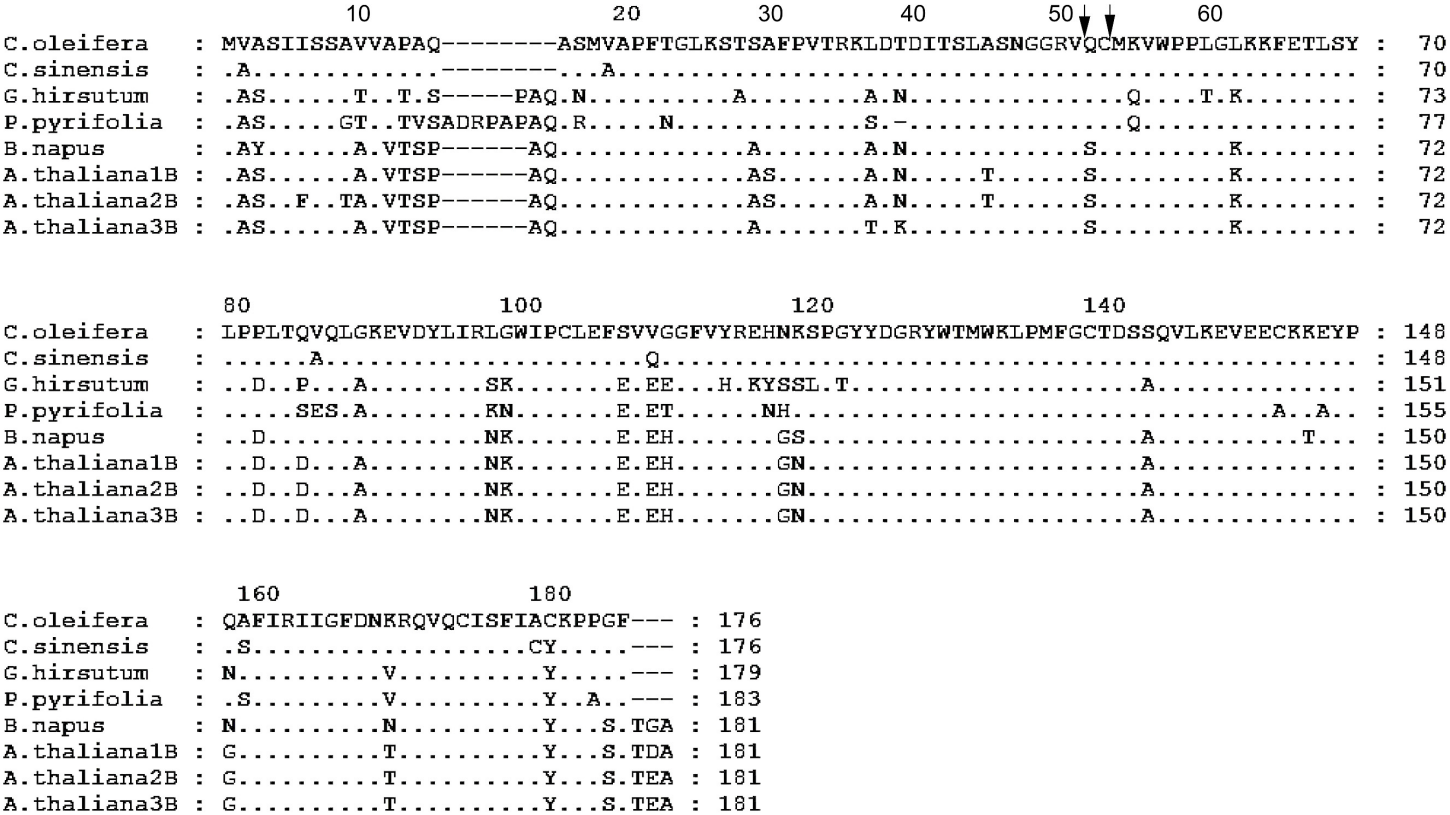
Alignment of the amino acid sequence of *rbcS* from *Camellia oleifera* with the sequence from *Camellia sinensis*, *Gossypium hirsutum*, *Pyrus pyrifolia*, *Brassica napus*, and *Arabidopsis thaliana*. *A. thaliana* 1B, 2B, and 3B represent *A. thaliana* ribulose bisphosphate carboxylase small chain 1B (NP_198659), *A. thaliana* ribulose bisphosphate carboxylase 2B small chain (NP_198658), and *A. thaliana* small chain 3B (NP_198657), respectively. Amino acid residues identical to those of *C. oleifera* rbcS protein are indicated by dots. Hyphens indicate gaps. The arrows indicate the possible processing site of mature protein in *C. oleifera*.

Two strong transmembrane helices, signal peptides or transmembrane regions were found in the putative polypeptide of Co-rbcS by SignalP 3.0 Server using neural networks (NNs) and hidden Markov models (HMMs) trained on eukaryotes (**Figure 6**). The most likely cleavage site appeared to be between positions 16 and 17. Acidic residues and basic residues account for 25.5 and 12.25%, respectively. Hydrophobic residues and charged residues are 28.90 and 18.89%, respectively. Alignment of the putative Co-rbcS amino acid sequence with rbcS from the other plants showed that Co-rbcS shared 96% homology with that of *C. sinensis* (ABK15574), 78% homology with that of *Gossypium hirsutum* (AFS41732) and *P. pyrifolia* var. culta (BAA00450) rbcS, and 77% homology with that of *B. napus* rbcS (ABB51649) and *A. thaliana* small chain 3B (NP_198657) as well as 75 and 76% homology with *A. thaliana* ribulose bisphosphate carboxylase 2B small chain (NP_198658) and *A. thaliana* ribulose bisphosphate carboxylase small chain 1B (NP_198659), respectively.

### Expression of *CO-rbcL* and *CO-rbcS* in *C. oleifera* Cultivars

**FIGURE 7:**
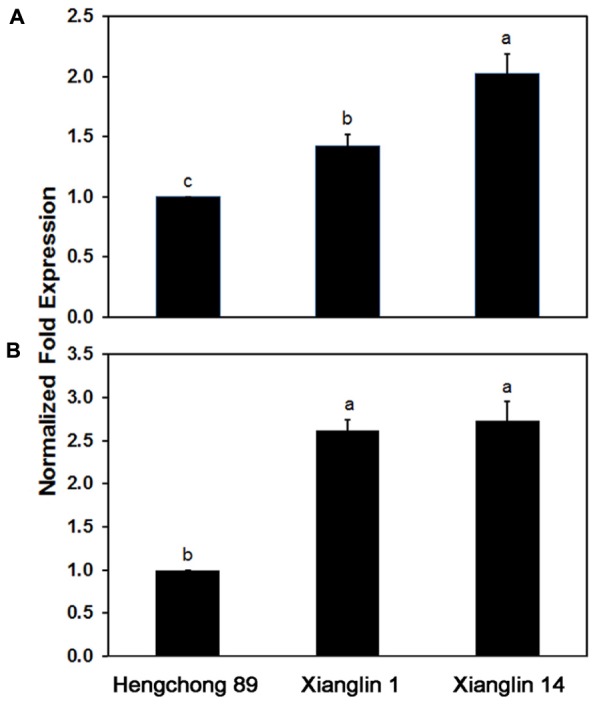
Expression of *Co-rbcL* **(A)** and *Co-rbcS* **(B)** in leaves of three cultivars Hengchong 89, Xianglin 1, and Xianglin 14 analyzed by real-time quantitative PCR, and the expression levels were normalized based on internal control of *GAPDH* gene. Bars represent standard error with *n* = 3. Different letters above the bar represent significant difference at *P* < 0.05 level by Duncan’s Multiple Range Test.

Real-time quantitative PCR analysis showed that *Co-rbcL* expression in cultivar Xianglin 14 was 42% higher than Xianglin 1 and 103% higher than Hengchong 89; *Co-rbcL* expression in Xianglin 1 was 41% higher than Hengchong 89 (**Figure 7A**). The expression levels of *Co-rbcS* in Xianglin 1 and Xianglin 14 were similar (**Figure 7B**) but were more than 2.5 fold higher than Hengchong 89.

### Photosynthesis

The mean net photosynthetic rate (*P_N_*) of cultivar Xianglin 14 from 3-year data (2011–2013) was 12.55 μmol CO_2_ m^-2^ s^-1^, which was the highest (**Table 1**). The *P_N_* of Xianglin 1 was significantly higher than Hengchong 89. Stomatal conductance (*g*_s_) of Xianglin 14 was also significantly higher than Xianglin 1, and Xianglin 1 was greater than Hengchong 89. On the other hand, intercellular CO_2_ concentration (*C*i) was significantly higher in ‘Hengchong 89’ than both ‘Xianglin 14’ and ‘Xianglin 1’. Transpiration rates (*E*) of ‘Xianglin 14’ and ‘Xianglin 1’ were similar but both were significantly higher than ‘Hengchong 89’.

**Table 1:** Net photosynthetic rate, *P*_N_ [μmol(CO_2_) m^-2^ s^-1^]; stomatal conductance, *g*_s_ [mol(H_2_O) m^-2^ s^-1^]; intercellular CO_2_ concentration, *C*_i_ [cm^3^ m^-3^]; and transpiration rate, *E* [mmol(H_2_O) m^-2^ s^-1^] of three *C. oleifera* cultivars determined from 2011 to 2013 when grown in Hunan, China.

Cultivar	*P*_N_	*g*_s_	*C*_i_	*E*
Hengchong 89	10.52 ± 0.70c^z^	0.11 ± 0.01c	225.75 ± 7.64a	2.65 ± 0.20b
Xianglin 1	11.46 ± 0.99b	0.12 ± 0.02b	212.55 ± 18.61b	3.35 ± 0.55a
Xianglin 14	12.55 ± 0.99a	0.13 ± 0.01a	209.67 ± 15.45b	3.63 ± 0.18a

*^z^Data were means of 3-year readings, and different letters within a column represent significant difference at *P* < 0.05 level by Duncan’s Multiple Range Test (*n* = 9)*.

### Relationships among Oil Yield, P_N_, and *CO-rbcL* and *CO-rbcS* Expression

Pearson’s correlation analysis showed that oil yield was significantly correlated with the expression levels of *Co-rbcL* and *Co-rbcS* at the *P* < 0.001 level (*r*= 0.94) and *P* < 0.01 level (*r*= 0.78), respectively (**Table 2**). The oil yield was also highly correlated with *P_N_* at the *P* < 0.001 level (*r*= 0.97). Furthermore, the expression level of *Co-rbcL* was closely correlated with dry seed yield at *P* < 0.001 level (*r*= 0.94) and fresh seed yield *P* < 0.01 level (*r*= 0.86); however, there was no significant correlation between *Co-rbcS* expression and fresh and dry seed yields. Additionally, *P_N_* was significantly correlated with dry seed yield but was not significant with fresh seed yield.

**Table 2:** Person’s correlation coefficients (*r*) of oil yield, dry seed and fresh seed yields in relation to the normalized fold expression of *Co-rbcL* and *Co-rbcS* and net photosynthetic rate (*P_N_*) in three cultivars of *C. oleifera*.

Yield	*Co-rbcL*	*Co-rbcS*	*P_N_*
Oil yield	0.94^∗∗∗^	0.78^∗∗^	0.97^∗∗∗^
Dry seed yield	0.94^∗∗∗^	0.42	0.74^∗^
Fresh seed yield	0.86^∗∗^	0.21	0.57

*^∗^, ^∗∗^, and ^∗∗∗^ represent significance at *P* < 0.05, *P* < 0.01, and *P* < 0.001 levels, respectively based on the table of critical values for Peason’s correlation coefficients (*r*) with df = 7.*

## Discussion

The primary photosynthetic CO_2_ reduction reaction, the binding of CO_2_ to the acceptor-molecule ribulose-1,5-bisphosphate (RuBP) to form two molecules of 3-phosphoglycerate, is catalyzed by the enzyme Rubisco (Andersson and Backlund, 2008). Rubisco is the most abundant soluble protein in the plant leaf (Ellis, 1979). Increasing photosynthesis has been shown to increase crop yield (Ainsworth and Long, 2005; Raines, 2011). Photosynthesis provides the source of carbon and the reducing power and ATP for *de novo* fatty acid synthesis, and increased carbons enhance the rate of fatty acid synthesis (Rawsthorne, 2002; Bates et al., 2013). In the search for potential molecular markers that might help improve *C. oleifera* breeding efficiency for high oil yields, Rubisco genes could be a subject of interest.

The present study isolated Rubisco subunits *rbcL* and *rbcS* genes from *C. oleifera*. As illustrated in **Figure 5**, rbcL amino acid sequence of *C. oleifera* is 100 and 99% homologous to its relatives *C. taliensis* (Yang et al., 2013) and *C. sinensis* (GenBank accession number: YP_007317256), respectively and is more than 90% homologous to other higher plant species, which supports the conclusion that the *rbcL* gene is highly conserved (Manzara and Gruissem, 1988). In addition to the identified active sites and transcription processing site in the *Co-rbcL*, the Co-rbcL protein also included eight cysteine residues at positions of 84, 172, 192, 221, 247, 284, 427, and 459. The Cys pairs 172 to 192 and 449 to 459 can create disulfide bridges within each LSU, while Cys 247 is thought to bridge between two adjacent subunits (Cohen et al., 2005). Residue Asp473 was proposed to serve as a latch responsible for placing the large-subunit carboxy-terminus over loop 6 and stabilizing the closed conformation required for catalysis (Duff et al., 2000); however, it is not essential for catalysis but for CO_2_/O_2_ specificity (Satagopan and Spreitzer, 2004). Lysine 334 is thought to play a specific role in stabilizing the transition state intermediates of both the carboxylation and oxygenation reactions, thereby facilitating the reaction of the enefiolate with the gaseous substrate (Parry et al., 2003). The amino acid sequence of rbcS of *C. oleifera* is only slightly above 75% homologous to the other plant species (**Figure 6**), indicating that *rbcS* is less conserved than *rbcL* (Manzara and Gruissem, 1988). Based on SOPMA prediction (Combet et al., 2000), the protein includes 22.16% of α helix, 24.43% of β sheet, 6.25% of β turn, and 47.16% of random coil. Moreover, the Co-rbcS precursors appeared to be processed to mature protein during entry into the chloroplast, and the processing site was postulated to occur between residues Gln(Q)^52^ and Cys(C)^53^ using ChloroP 1.1 Prediction Server^6^.

Three-year field studies showed that the highest seed yield was produced by the *C. oleifera* cultivar Xianglin 14 (**Figure 2A**), which also produced the highest amount of oil (**Figure 2B**). Hengchong 89 had the lowest seed yield and was also the lowest in oil production. Xianglin 1 had a similar seed yield as Hengchong 89; however, its oil yield was significantly greater than Hengchong 89, suggesting that long-term selection for oil yield may result in cultivars also differing in fatty acid biosynthesis. Subsequent real-time quantitative PCR analysis showed that the level of *Co-rbcL* expression in Xianglin 14 was substantially higher than Xianglin 1; and Xianglin 1 was significantly greater than Hengchong 89 (**Figure 7B**). The expression levels of *rbcS* in Xianglin 14 and Xianglin 1 were similar, but their expressions were much greater than Hengchong 89 (**Table 1**). Examining leaf photosynthesis showed that *P_N_* were significantly higher in Xianglin 14 than Xianglin 1; and Xianglin 1 was higher than Hengchong 89 (**Table 2**). Pearson’s correlation analysis demonstrated that *Co-rbcL* expression levels were significantly correlated with oil and dry seed yields at *P* < 0.001 level as well as with fresh yield at *P* < 0.01 level; while *Co-rbcS* expression was only correlated with oil yield at *P* < 0.01 level. The *P_N_* was correlated with oil yields at *P* < 0.001 level and dry seed yields at *P* < 0.05 level (**Table 2**). Our results suggest that *Co-rbcL* and *Co-rbcS*, particularly *Co-rbcL*, could potentially be molecular markers for early selection of higher oil producing cultivars. Furthermore, the selection should also take the net photosynthetic rate into consideration. The incorporation of *Co-rbcL* and *Co-rbcS* markers into breeding programs could allow early recognition of potential high oil production cultivars and could significantly shorten tea oil plant breeding time and increase breeding efficiency.

The interest in the relationships of Rubisco activity, Rubisco proteins, and/or their mRNAs with crop yield dates back to the 1970s (Frey and Moss, 1976; Murthy and Singh, 1979). Some contradictory reports on the relationship of photosynthesis and crop yield in company with suggestions that the crop is a sink and not photosynthesis limited (Kuo et al., 1980; Loza-Tavera et al., 1990; Evans, 1998; Borras et al., 2004) have led to the view that improving photosynthesis is unlikely to increase crop yield (Borras et al., 2004; Sinclair et al., 2004). Information regarding improving photosynthesis to enhance crop yield became limited, even though traditional breeders have been continuously working on the improvement of photosynthesis and other economically important traits for yield increase. The reappraisal of the importance of photosynthesis for yield enhancement has been a more recent event (Long et al., 2006; Fischer and Edmeades, 2010; von Caemmerer and Evans, 2010), which is based on at least three lines of evidence: first, leaf photosynthesis increase resulted from CO_2_ enrichment generally leads to increased crop yield. Second, C_4_ plants have greater rates of photosynthesis and produce more biomass per unit of intercepted sunlight than C_3_ plants. Third, the introduction of dwarf genes into rice and wheat for effectively capturing light significantly increased the fraction of biomass in grain. Several recent publications have documented photosynthesis improvement in enhancing corn, rice, soybean, and wheat yields (Long et al., 2006; Fischer and Edmeades, 2010; Zhu et al., 2010; Parry et al., 2011; Ainsworth et al., 2012). Phylogenetic analysis also showed that positive selection in the *rbcL* gene of higher plants is common (Kapralov and Filatov, 2007), and Rubisco kinetics is quite variable among plant species (Flood et al., 2011; Galmes et al., 2014). Furthermore, two transplastomic tobacco lines with functional Rubisco from the cyanobacterium *Synechococcus elongatus* had higher rates of CO_2_ fixation per unit of enzyme than the control plant (Lin et al., 2014), suggesting that Rubisco activity and regulation should be targets for improving plant productivity.

We consider our study as a part of the effort on the recognition of the importance of photosynthesis for yield improvement. The continuing and long-term selection of high seed and high oil yields in *C. oleifera* could lead to enrich the abundances of transcripts of Rubisco genes that enhance photosynthetic properties, photosynthetic efficiency or capacity. Since the foremost breeding goal has been selecting cultivars with high oil production, the selective action may also enhance fatty acid biosynthesis, thus improving crop oil yield (Long et al., 2006; Zhu et al., 2010; Parry et al., 2011). This could be due to the fact that Rubisco determines photosynthetic efficiency, and photosynthesis is the source of carbon and reducing power and ATP for *de novo* fatty acid synthesis. The present study may also suggest that genes related the biosynthesis of oil could be enriched by the selection (Zeng et al., 2014). In addition to investigating mechanisms underlying the enhanced *Co-rbcL* and *Co-rbcS* expression, further studies are warranted to examine if some oil biosynthesis genes are also highly expressed in ‘Xianglin 14’. Nevertheless, the identified Rubisco genes, particularly *Co-rbcL* could potentially be molecular markers for improving breeding efficiency in *C. oleifera*.

## Conclusion

The present study is the first report of *Co-rbcL* and *Co-rbcS* in *C. oleifera*, a tree species producing high-quality edible oil. Analyzing the gene expression and net photosynthetic rates of three cultivars differing in oil production showed that oil yields were highly correlated with the expression levels of *Co-rbcS* and *Co-rbcL* in particular as well as net photosynthetic rates. These results suggest that *Co-rbcL* and *Co-rbcS* could potentially be molecular markers for early selection of high oil production cultivars of *C. oleifera*.

## Author Contributions

YC, BW, and JC conceived and designed the experiments. BW, XW, RW, SP, LC, LM, and JL conducted the experiments. All the authors participated in the analysis and interpretation of the results. YC, BW, and JC wrote this manuscript, which was approved by all authors.

## Footnotes

1 http://www.ncbi.nlm.nih.gov

2 http://www.expasy.ch/tools/protparam.html

3 http://www.ch.embnet.org/software/TMPRED_form.html

4 http://www.cbs.dtu.dk/services/SignalP/

5 http://npsa-pbil.ibcp.fr/cgi-bin/npsa_automat.pl?page=/NPSA/npsa_sopma.html

6 http://www.cbs.dtu.dk/services/ChloroP/

7 http://primer3.ut.ee/
